# Stem cells set their sights on retinitis pigmentosa

**DOI:** 10.7554/eLife.01291

**Published:** 2013-08-27

**Authors:** Jeannette L Bennicelli, Jean Bennett

**Affiliations:** 1 **Jeannette L Bennicelli** is at the F.M. Kirby Center for Molecular Ophthalmology, University of Pennsylvania School of Medicine, Philadelphia, United Statesjbennice@mail.med.upenn.edu; 2 **Jean Bennett** is at the F.M. Kirby Center for Molecular Ophthalmology, University of Pennsylvania School of Medicine, Philadelphia, United Statesjebennet@mail.med.upenn.edu

**Keywords:** ophthalmology, retinal degeneration, induced pluripotent stem cells, retinal transplantation, retinal cell differentiation, retinitis pigmentosa, Human, Mouse

## Abstract

Skin cells from a patient with a form of inherited blindness have been reprogrammed into retinal cells and successfully transplanted into mice.

**Related research article** Tucker BA, Mullins RF, Streb LM, Anfinson K, Eyestone ME, Kaalberg E, Riker MJ, Drack AV, Braun TA, Stone EM. 2013. Patient-specific iPSC-derived photoreceptor precursor cells as a means to investigate retinitis pigmentosa. *eLife* **2**:e00824. doi: 10.7554/eLife.00824

**Image** Photoreceptors derived from human stem cells can colonize a mouse retina (arrow)

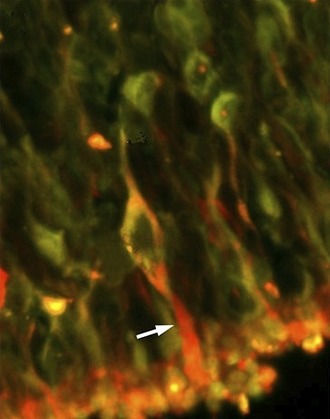

Inherited blindness encompasses a wide spectrum of pathologies that can be caused by mutations in more than 220 genes. Retinitis pigmentosa is one of the most common forms of the 250 known types of heritable vision loss, and is characterized by progressively diminishing sight due to the death of cells within the retina. It occurs in both genetically dominant and recessive forms, and results from hundreds of distinct mutations within dozens of genes. Although some treatments are available, the high level of idiosyncrasy associated with the condition has led researchers to conclude that stem-cell–based approaches might provide the best hope for a cure.

To explore the possibility of treating inherited blindness with stem cells, Edwin Stone and co-workers at the University of Iowa routinely screen DNA from individuals experiencing vision loss to identify the genetic mutations leading to their blindness; the Iowa team also generate induced pluripotent stem cells (iPSCs) from these individuals to create patient-specific models of disease. Now, in *eLife*, Stone and co-workers—including Budd Tucker as first author—report that they have used stem cell technology to create a personalized model of a recessive form of retinitis pigmentosa, and that they have also successfully transplanted the cells into mice (Tucker et al., 2013). These results are an important step toward autologous transplantation, the regeneration of tissues damaged by disease using stem cells derived from the patient’s own cells (Figure 1). In addition to benefiting basic research, these findings represent a means to develop specific understanding of, and treatment for, a range of genetic conditions—in particular, the large set of highly idiosyncratic syndromes that constitute inherited blindness.

**Figure 1.**
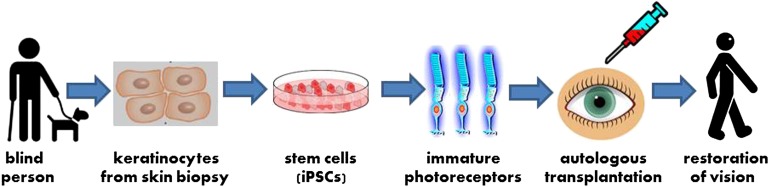
A new strategy for autologous cell transplantation to treat inherited blindness. A skin biopsy from a patient with retinitis pigmentosa—a heritable form of blindness that results from the loss of photoreceptor cells in the retina—is used to isolate keratinocytes that are then reprogrammed into induced pluripotent stem cells (iPSCs). The iPSCs are stimulated to differentiate into precursors to photoreceptor cells in a petri dish. These immature photoreceptor cells can be assessed in culture to identify the defects that resulted in the patient’s blindness (not shown). They can also be used for gene therapy and transplanted into the patient’s eye. Successful transplantation is followed by photoreceptor maturation and restoration of functional vision.

iPSCs were first generated in 2007 by reprogramming human fibroblast cells isolated from the skin, and were shown to approximate embryonic stem cells (Takahashi et al., 2007; Yu et al., 2007). The most important feature of iPSCs and embryonic stem cells is that they are ‘pluripotent’: in other words, they have the capacity to differentiate into many distinct mature cell types normally found in the body. Since iPSCs can be created by reprogramming the cells of adults (as opposed to embryonic stem cells, which, as their name suggests, are derived from developing embryos), they provide a means to precisely model a single individual’s genetic disease. The cells can also be used to screen potential treatments for efficacy.

iPSCs also pave the way toward gene therapy. For example, retinal cells can be generated in culture from patient-derived iPSCs and used to study whether the introduction of specific genes into these cells can compensate for a biological defect in their function. Furthermore, disease-causing mutations can be corrected outside of a patient’s body by ‘editing’ the DNA within the cells, and the corrected cells can then be re-differentiated to form the type of cell destroyed by the disease. Ultimately, researchers hope to be able to introduce either specific genes or healthy, mature cells derived from iPSCs into a diseased tissue to restore normal function.

The Iowa team have now made progress toward understanding, and perhaps even treating, a form of autosomal recessive retinitis pigmentosa (ARRP). The study involved a blind 62-year-old male who attended a vision clinic at their institution. The first step was to identify the mutations causing the disease; these mutations were found to be in *USH2A*, a gene that has previously been associated with both ARRP and Usher syndrome (which causes blindness and deafness). Mutations were identified in both alleles of the gene, including one in a non-coding region, as also recently reported by other researchers (Vache et al., 2012).

Stone and co-workers next generated iPSCs from cells taken from the patient’s skin. By stimulating these iPSCs to differentiate, they were able to obtain various retinal cell types as reported by others (Meyer et al., 2011; Rowland et al., 2012), and moreover the cells formed structures similar to retinal ‘eyecups’. Initially these eyecups contained retinal pigment epithelium, a tissue that supplies nutrients to photoreceptors. Over time, photoreceptor cells also began to develop; these cells were identified by the presence of the visual pigment rhodopsin, as well as various structural characteristics. Importantly, the patient’s iPSC-derived tissues offered a model for the onset of the patient’s retinitis pigmentosa: while the mutations did not impair the development of the eyecups in culture, the mutated USH2A protein was misfolded, resulting in cellular stress that likely caused the death of photoreceptors, and hence blindness.

Whether cells grown in vitro can colonize a tissue in vivo will be crucial to the medical application of this work. To assess how their iPSC-derived cells would fare in live tissue, the Iowa team transplanted the immature photoreceptor cells into the eye of a mouse. Within two weeks these cells had integrated into the retina and had differentiated, indicating that this method might also have favourable prospects in human patients with inherited blindness.

The workflow described by Stone and co-workers is particularly noteworthy given the patient’s age. Their success was likely facilitated by the decision to use keratinocytes from the initial skin biopsy, instead of another cell type found in skin, fibroblasts. Cells from older individuals are much harder to reprogram than those from younger ones, and it is both faster and 100-fold more efficient to reprogram keratinocytes than fibroblasts (Aasen et al., 2008). The Iowa team chose to work with keratinocytes since both they and retinal tissue are derived from the same embryonic tissue (ectoderm), reasoning that differentiation of iPSCs into retinal tissue might be facilitated by the use of cells with a similar origin. Also, keratinocyte-derived iPSCs have been reported to resemble embryonic stem cells more than those derived from fibroblasts (Barrero et al., 2012), and promising petri dish models of 3-dimensional eyecup formation have been generated using embryonic stem cells from human (Nakano et al., 2012) and mouse (Eiraku et al., 2011; Gonzalez-Cordero et al., 2013). Notably, eyecups did not form in previous studies where these researchers used fibroblasts to make iPSCs.

The Iowa team’s ultimate goal is to develop a patient-specific therapy using photoreceptor cells generated from iPSCs. In the case of *USH2A*, gene therapy to introduce a ‘clean copy’ of the gene is likely to be a poor solution, as the coding region of this gene is too large for established retinal gene therapy vectors. Further, many of the photoreceptor cells in the retina of this particular patient had already died due to disease, and therefore could not respond to gene therapy. In cases such as this, cell transplantation seems the most viable strategy, and the results reported by Stone and co-workers after transplantation of immature photoreceptor cells in mice are promising. This patient—and others with similar mutations—could potentially benefit for decades if the cells were to integrate and differentiate appropriately in his retina, since he had reasonable vision until his third decade of life. An even longer-lasting effect could result if one or both of the patient’s *USH2A* mutations were corrected, prior to transplantation, in iPSC-derived photoreceptor precursors using gene editing approaches.

Many other studies will clearly be needed before this method can be tested in humans, including optimization of the parameters for transplantation; evaluation of the safety and longevity of the transplanted cells; and demonstration of the ability of the transplanted cells to adequately restore vision. The studies from Stone and co-workers are, however, an exciting step forward and provide the groundwork for new approaches for treating blindness using personalized cell models.
